# Optimization of Molecularly Imprinted Polymer Method for Rapid Screening of 17*β*-Estradiol in Water by Fluorescence Quenching

**DOI:** 10.1155/2011/214747

**Published:** 2011-06-16

**Authors:** Yu Yang, Edward P. C. Lai

**Affiliations:** Department of Chemistry, Carleton University, Ottawa, ON, Canada K1S 5B6

## Abstract

A new method was optimized for rapid screening of 17*β*-estradiol (E2) in water under 10 min. Molecularly imprinted polymer (MIP) particles (325 ± 25 nm) were added in a water sample at pH 5.5 and 20°C to form a suspension. Fluorescence emission from E2 nonspecifically bound onto the MIP particles was first quenched by large gold nanoparticles (43 ± 5 nm). The Stern-Volmer plot was linear, with dynamic quenching constants (*K*_sv_) of 2.9 ×10^4^ M^−1^. Fluorescence emission from E2 specifically bound inside the MIP particles was next quenched by small nitrite anions that easily penetrated the imprinted cavities. The Stern-Volmer plot became nonlinear, with *K*_sv_ = 2.1 × 10^2^ M^−1^ and static quenching constant (V) below 1.0 M^−1^. The difference between these two emission intensities varied as the initial E2 concentration in water, generating a Scatchard calibration curve with *R* ^2^ > 0.97 from 0.1 to 10 ppb.

## 1. Introduction

Molecularly imprinted polymers (MIPs) exhibiting high selectivity and affinity to the target molecule are well recognized as a fast growing research field [1, 2]. They have been successfully applied in various novel methods of chemical analysis [3, 4], including potentiometric sensors [5–7], amperometric detection [8], and differential pulse cathodic stripping voltammetry [9]. An optical sensor was fabricated with an MIP film for the determination of formaldehyde molecules that induced measurable optical reflectivity shifts [10]. For surface plasmon resonance spectroscopy, MIP particles were spin-coated onto a gold surface to detect theophylline [11]. MIP fibers were used in a sensing device to determine folic acid [12].

**Scheme 1:**
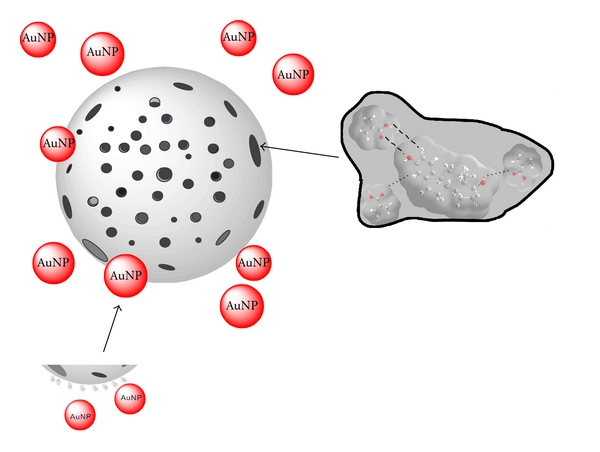
Larger AuNPs were used in the first step of fluorescence quenching to quench E2 molecules nonspecifically bound throughout the porous MIP particle while small nitrite anions easily penetrate the MIP particle to quench the fluorescence emission from E2 molecules specifically bound inside imprinted cavities in the second step of fluorescence quenching. The attenuated fluorescence emission intensity between step 1 and step 2 varies as a function of E2 concentration.

A novel sensing scheme based on nonlinear fluorescence quenching of 17*β*-estradiol (E2) was recently developed in our laboratory [13]. Small nitrite ions penetrated the porous structure of MIP particles and quenched the fluorescence emission from E2 molecules inside imprinted cavities. On the contrary, large methacrylamide molecules (3-D stearic diameter = 0.536 nm) were hindered when penetrating the pores to access the imprinted cavities, resulting in low dynamic quenching. Research was continued in our laboratory to evaluate larger quenchers, such as gold nanoparticles (AuNPs) that could readily be synthesized with a diameter of 43 ± 5 nm [14–16]. Their effectiveness was studied with regard to quenching the fluorescence of only nonspecifically bound E2 molecules throughout the porous MIP particles, but not those specifically bound inside the imprinted cavities, as illustrated in Scheme 1. The objective of this work was to develop a rapid method (hopefully under 5 min) for the determination of trace E2 in water (down to 0.1 ppb). 

## 2. Experimental Section

### 2.1. Chemicals

Sodium citrate tribasic dihydrate, gold (III) chloride trihydrate, sodium nitrite, and E2 were purchased from Sigma-Aldrich (St. Louis, MO, USA). Methacrylic acid and methacrylamide were purchased from Aldrich (Milwaukee, WI, USA). 2,2-azobisisobutyro-nitrile (AIBN) was purchased from Pfaltz and Bauer (Waterbury, CT, USA). Methanol (HPLC grade), acetonitrile (HPLC grade), and acetone (spectro grade) were purchased from Caledon (Georgetown, ON, Canada). Acetic acid (reagent grade) was purchased from Anachemia (Montreal, QC, Canada). 18-MΩ·cm distilled deionized water (DDW) was obtained from a Millipore Milli-Q water system (Bedford, MD, USA). 

### 2.2. Preparation of MIP Submicron Particles and AuNPs

The method for preparation of E2 MIP submicron particles had previously been described [17]. These particles (80 mL) were washed with 15% acetic acid in methanol (v/v), methanol, acetonitrile, and DDW three times. Each washing was combined with 1 hr of sonication and 1 hr of centrifugation to completely extract template E2 molecules from the particles and remove polymerization reagent residues. After the last washing with DDW, the pH was 5.5 ± 0.1 in the supernatant and the free E2 concentration was below the detection limit of HPLC-FD instrument. These washed MIP submicron particles were dried at 70°C. Another batch of freshly prepared MIP submicron particles was washed only with DDW for 25 times. These washed E2-MIP particles would contain the maximum E2 loading [13]. AuNPs were synthesized by adapting a previously reported method [18]. No washing was applied to these AuNPs. 

### 2.3. Particle Size Analysis

The AuNPs, MIP, and E2-MIP particles were suspended in 10 M KNO_3_ at a concentration of 40 mg/mL. The suspensions were sonicated for 15 min before measurement on a NanoDLS particle size analyzer (Brookhaven Instruments, Holtsville, NY, USA). The instrument had been calibrated by 92 ± 4 nm Nanosphere size standards (Duke Scientific, Palo Alto, CA, USA). A total of 10 measurements were run after 30 s of quiescence time, and the laser beam intensity was automatically optimized by the analyzer before each run. 

### 2.4. Fluorescence Quenching

3.5 ppm E2 (2 mL) and 2.5 mg/mL E2-MIP particles (2 mL) were added into two 3-mL quartz cuvette cells, each equipped with a polytetrafluoroethylene (PTFE) stopper. Then 1 mL of AuNPs aqueous suspension with elemental concentrations from 0 M to 5.88 × 10^−4^ M was used to quench the E2 and E2-MIP particles fluorescence emission intensities. All emission intensities were measured at room temperature (20 ± 1°C) by a fluorescence spectrophotometer (Varian Cary Eclipse, Palo Alto, CA, USA) using excitation wavelength of 280 ± 1 nm and emission wavelength of 310 ± 1 nm (or scanning from 290 nm to 450 nm). Both the excitation and emission slits were set at 5 nm. To test for inner filter effect, 1 mL of 1% (w/w) trisodium citrate dihydrate was used instead of AuNPs. Similarly, E2 and E2-MIPs fluorescence quenching experiments with sodium nitrite were accomplished under exactly the same experimental conditions. 

Two-step fluorescence quenching by first AuNPs and then sodium nitrite was performed. 7.7 ± 0.1 mg, 5.5 ± 0.1 mg, 3.3 ± 0.1 mg, and 1.1 ± 0.1 mg of template-removed MIP submicron particles were added into 2.2 mL of E2 aqueous solution with concentrations from 0.0001 ppm to 3.5 ppm. The blank and E2-templated MIP particles were prepared by using the same amount of template-removed MIPs and E2-MIP particles suspended in 2.2 mL of DDW. All of these suspensions were incubated under sonication for 5–35 min at room temperature (20 ± 1°C). Then, 2.0 mL of E2-bound MIP or E2-MIP submicron particle suspension was transferred into a 3 mL quartz cuvette cell and spiked with 1 mL of 5.88 × 10^−4^ M AuNPs aqueous suspension. After the fluorescence emission intensity was recorded, 100 *μ*L of 150 ± 1 mg/mL sodium nitrite was added to perform the second step of fluorescence quenching. 

All light absorption spectra by quenchers were measured on a UV-visible spectrophotometer (Varian Cary 3, Palo Alto, CA, USA) by scanning from 250 nm to 350 nm at room temperature (20 ± 1°C) to investigate the inner filter effect. The absorption of both exciting light (*λ*_ex_ = 280 ± 1 nm) and fluorescence emission (*λ*_em_ = 310 ± 1 nm) by quenchers was corrected, for right-angle illumination, as described elsewhere [19]. 

## 3. Results and Discussion 

### 3.1. Fluorescence Quenching

**Figure 1:**
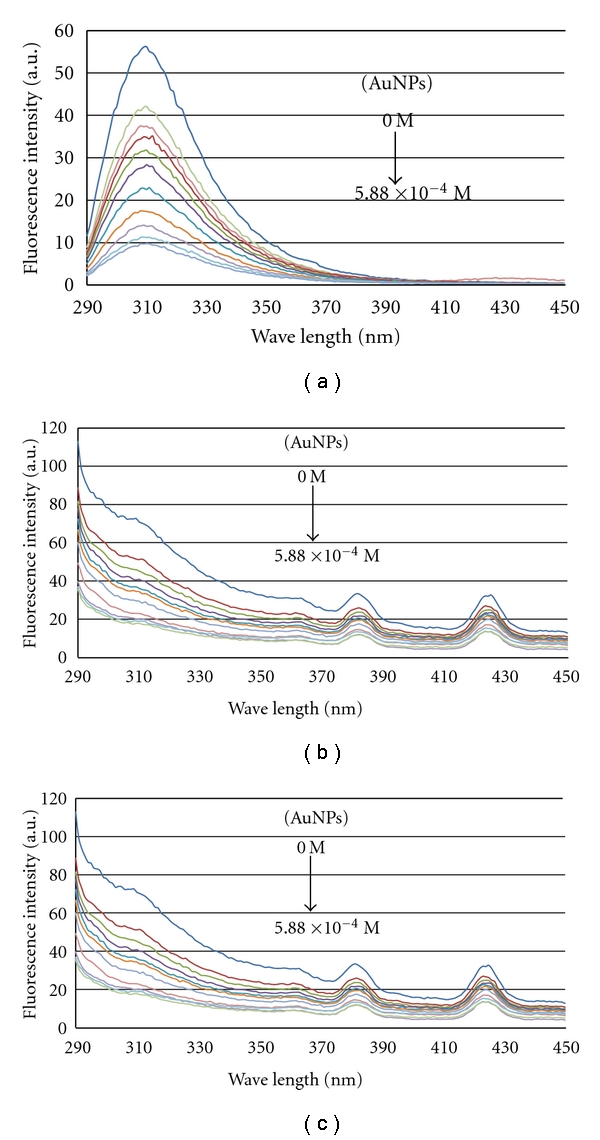
Fluorescence emission spectra of (a) 3.5 ppm E2 aqueous solution, (b) 2.5 mg/mL E2-MIP particles in aqueous suspension, and (c) 2.5 mg/mL E2-bound NIP particles in aqueous suspension, during fluorescence quenching by AuNPs from 0 M to 5.88 × 10^−4^ M. (*λ*_ex_ = 280 nm).

E2 is a naturally fluorescent compound. After it interacts with nonfluorescent MIP particles both specifically and nonspecifically [20], the bound E2 molecules can be determined by spectrofluorimetry (*λ*_ex_ = 280 nm and *λ*_em_ = 310 nm) [21].  Figure 1 shows the fluorescence emission spectra of E2, E2-MIP particles, and E2-bound NIP particles during their quenching by AuNPs. Without particles, a 3.5 ppm E2 aqueous solution exhibited decreasing fluorescence intensities when AuNPs were added stepwise as shown in Figure 1(a). The fluorescence intensity decreased by almost 82% from the initial level as the concentration of AuNPs reached 5.88 × 10^−4^ M. Similarly, the quenching effects of AuNPs on E2-MIP particles and E2-bound NIP particles are evidenced in Figures 1(b) and 1(c), decreasing the fluorescence intensity by 76% and 77%. The strong Mie scattering peak (at 280 nm) from particles slightly enhanced all E2 fluorescence emission peaks at 310 nm, which can be considered meritorious for the determination of E2 at trace levels. Two Raman scattering peaks (at 380 nm and 425 nm) were characteristic of particles when an excitation wavelength of 280 nm was used. Luckily, they did not have any significant impact on the fluorescence quenching results. 

**Figure 2:**
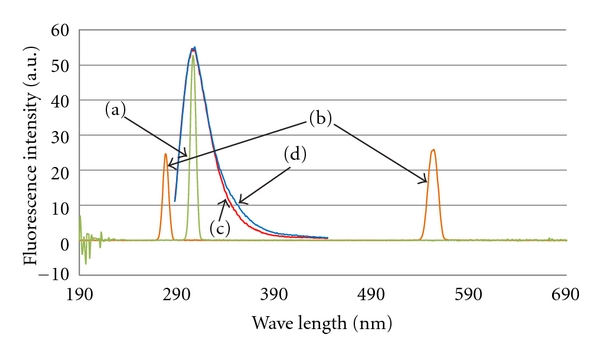
(a) Fluorescence excitation spectrum of AuNPs aqueous suspension (*λ*_em_ = 310 nm), (b) fluorescence emission spectrum (first and second orders) of AuNPs aqueous suspension (*λ*_ex_ = 280 nm), (c) fluorescence emission spectrum of E2 aqueous solution (*λ*_ex_ = 280 nm), and (d) fluorescence emission spectrum of E2 aqueous solution in presence of trisodium citrate dihydrate (1% w/w) (*λ*_ex_ = 280 nm).

The fluorescence properties of AuNPs were studied before they were used as a large fluorescence quencher in all subsequent experiments. When 5.88 × 10^−4^ M of AuNPs were examined by scanning the excitation wavelength in Figure 2(a) and using an emission wavelength of 310 nm, only one Mie scattering peak was observed at 310 nm. When the emission wavelength was scanned in Figure 2(b) using an excitation wavelength of 280 nm, only two Mie scattering peaks were found at 280 nm (first order) and 570 nm (second order). Therefore the AuNPs were nonfluorescent, making them ideal for use as fluorescence quencher in this work. Figure 2(c) shows the fluorescence emission spectrum of 3.5 ppm E2 aqueous solution while Figure 2(d) shows the same spectrum after addition of trisodium citrate dehydrate (1% w/w). No significant inner filter effect was observed from 1% trisodium citrate dihydrate, which was present in the synthesis of AuNPs. Furthermore in real samples, E2 may exist with some metabolites or other related compounds that can fluorescence. However, no interferences would be possible because these other fluorescent compounds cannot bind with the MIP cavities. Therefore after fluorescence quenching by AuNPs, the interferences can be eliminated.

**Figure 3:**
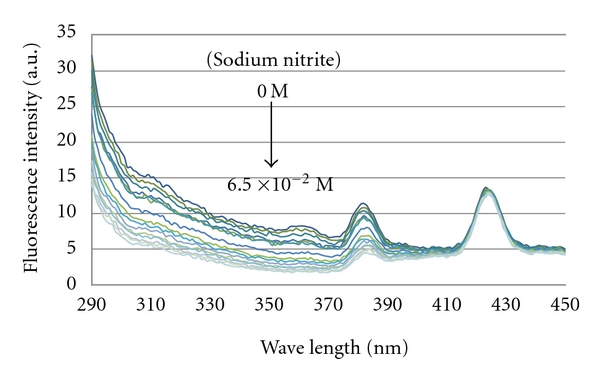
Fluorescence emission spectra during titration of sodium nitrite (up to a final concentration of 6.5 × 10^−2^ M) into a mixture of 2.5 mg/mL E2-MIP particles and 5.88 × 10^−4^ M AuNPs. The titration consisted of fifteen 10-*μ*L spikes of 100 mg/mL sodium nitrite to minimize any dilution effect (~6%).

In our previous study [13], sodium nitrite was able to quench the fluorescence emissions from E2 aqueous solution, E2-MIP aqueous suspension, and E2-bound NIP aqueous suspension. Hence, it was used in this work to finish the fluorescence quenching job after AuNPs quenched only the fluorescence emission from E2 that were nonspecifically bound inside particles. As shown in Figure 3, the residual fluorescence emission from 2.5 mg/mL E2-MIP particles in aqueous suspension, after quenching with 5.88 × 10^−4^ M AuNPs, was an intensity of 14.2 ± 0.2 arbitrary units (a.u.) coming mostly from E2 specifically bound inside the MIP cavities. Sodium nitrite was then titrated, stepwise from 0 M to 6.5 × 10^−2^ M, into the mixture of E2-MIP particles and AuNPs. Due to its small size, the nitrite anion penetrated through the porous MIP particles and quenched the fluorescence emission from the specifically bound E2 molecules. At the end of titration, the emission intensity was reduced to 5.5 ± 0.2 a.u. This result clearly demonstrated how simple it was to determine the amount of specifically bound E2 molecules. 

**Figure 4:**
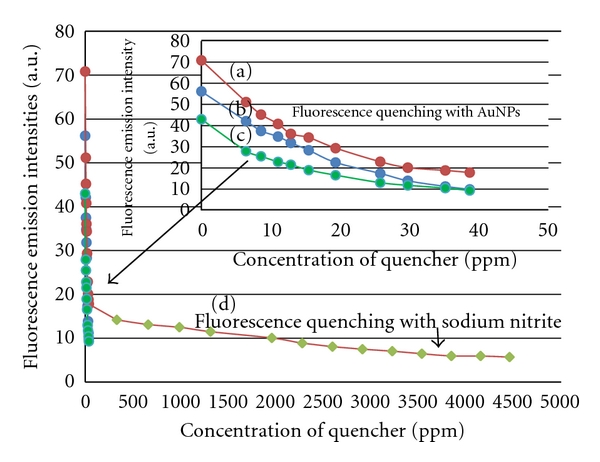
Fluorescence emission intensity at 310 nm versus concentration of quencher: (a) 2.5 mg/mL E2-MIP particles in aqueous suspension quenched with AuNPs, (b) 3.5 ppm E2 aqueous solution quenched with AuNPs, (c) 2.5 mg/mL E2-bound NIP particles in aqueous suspension quenched with AuNPs, (d) 2.5 mg/mL E2-MIP particles in aqueous suspension going through two steps of fluorescence quenching, first with AuNPs and next with sodium nitrite (standard error bars, approximately three-times the size of each data point symbol, are omitted here for the sake of clarity).

Figure 4 plots all the fluorescence emission intensities measured (at 310 nm) from Figures 1 and 3. Intuitively, both free E2 molecules in aqueous solution and nonspecifically bound E2 molecules in NIP particles (which had no imprinted cavities) in aqueous suspension were all accessible by AuNPs to undergo collisional quenching. If there were no imprinted cavities in MIP particles to protect the specifically bound E2 molecules (from quenching by AuNPs), the final emission intensity in Figure 4(a) would probably be as low as the ~10 a.u. in Figures 4(b) and 4(c) plots when the AuNPs quencher concentration reached 38.7 ppm (= 5.88 × 10^−4^ M. In reality, the E2-MIP particles contained some inaccessible E2 molecules that contributed to a higher final emission intensity of ~18 a.u. 

### 3.2. Quencher Sizes and Efficiencies

**Figure 5:**
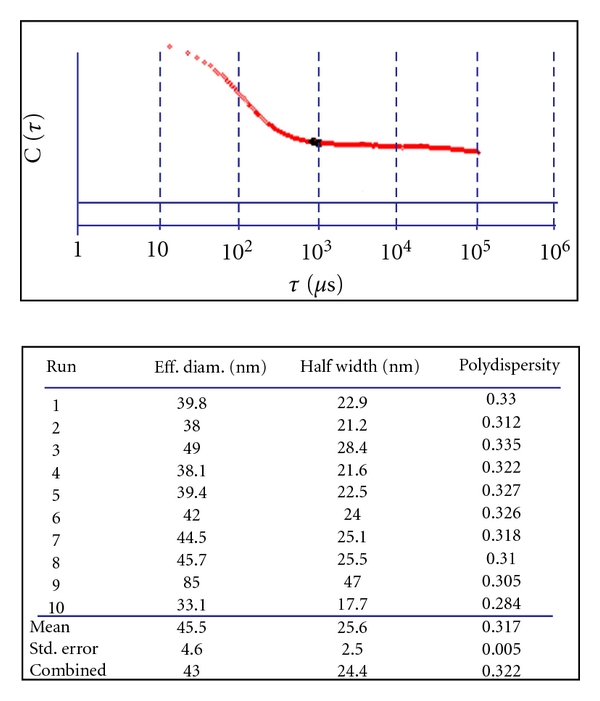
Correlation of light scattering intensity with time for measuring the size of AuNPs on a nanoDLS particle size analyzer.

The MIP and NIP particles studied in this work had diameters, as measured by a nanoDLS particle size analyzer, of 477 ± 11 nm and 373 ± 21 nm, respectively. E2 molecules were specifically bound inside the MIP cavities that were complementary in size, shape, and arrangement of functional groups. Small nitrite anions could easily penetrate the porous MIP particles and quench the fluorescence from the E2 molecules by dynamic collisions. The large AuNPs used in this study had a diameter of 43 ± 5 nm.  Figure 5 shows the correlation of light scatting intensity with time, as obtained for AuNPs during a particle size measurement. Over 10 runs, the particle size readings varied between 33 nm and 85 nm with a polydispersity of 0.3 (moderate dispersion). The size range seemed to be suited for fluorescence quenching of E2 molecules that were nonspecifically bound to MIP particles.

**Figure 6:**
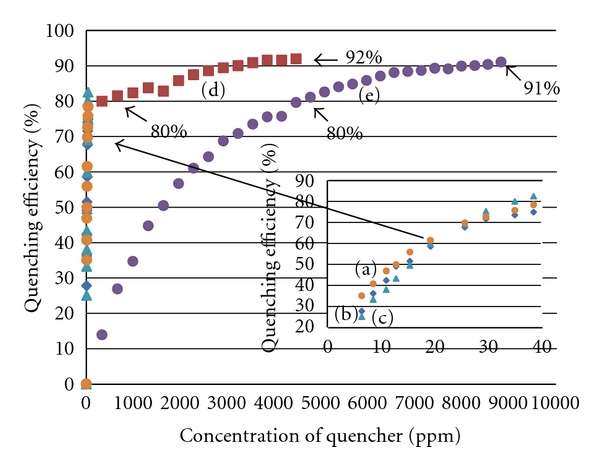
Quenching efficiency (*θ*) versus concentration of quencher: (a) 2.5 mg/mL E2-bound NIP particles in aqueous suspension quenched with AuNPs, (b) 2.5 mg/mL E2-MIP particles in aqueous suspension quenched with AuNPs, (c) 3.5 ppm E2 aqueous solution quenched with AuNPs, and (d) 2.5 mg/mL E2-MIP particles in aqueous suspension quenched first with AuNPs and next with sodium nitrite. (e) 2.5 mg/mL E2-MIP particles in aqueous suspension quenched with sodium nitrite (up to 0.128 M).

Figure 6 plots the fluorescence quenching efficiency (*θ* = 1 − *F*/*F*_0_, where *F* and *F*_0_ are the fluorescence emission intensities measured in the presence and absence of quencher) versus the concentration of quencher. With AuNPs, similar quenching efficiencies were observed for both particles and E2 in (a), (b), and (c). By comparison, sodium nitrite exhibited significantly lower quenching efficiency in (d) and (e). Approximately 4500 ppm sodium nitrite was needed in (e) to quench 80% of fluorescence emission from E2-MIP particles although only 38.7 ppm AuNPs was needed in (b). Interestingly a lesser amount of sodium nitrite was needed in the presence of AuNPs in (d) to quench E2-MIP particles, from 80% to 90%, than in (e). 

### 3.3. Stern-Volmer Plots

All fluorescence quenching data were analyzed further by applying the Stern-Volmer (S-V) equations that examine different quenching mechanisms [18, 22]:

(1) $$\begin{array}{c} \frac{F_{0}}{F}=1+K_{\text{sv}}\left[Q\right], \end{array}$$

(2) $$\begin{array}{c} \frac{F_{0}}{F}=\left(1+K_{\text{sv}}\left[Q\right]\right)\exp\;\;\left(V\left[Q\right]\right) \end{array}.$$

**Figure 7:**
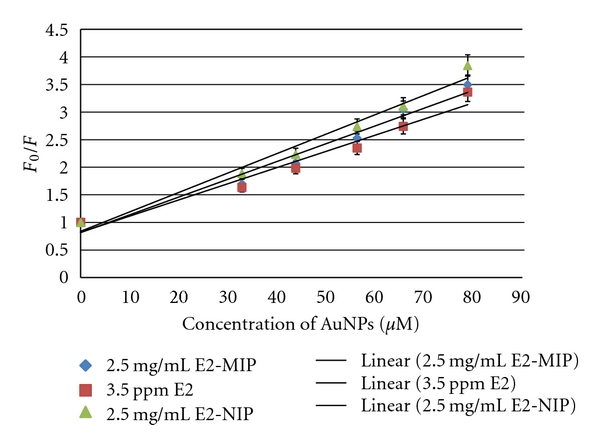
Stern-Volmer plots of *F*_0_/*F* versus concentration of AuNPs for 3.5 ppm E2 aqueous solution, 2.5 mg/mL E2-MIP particles in aqueous suspension, and 2.5 mg/mL E2-bound NIP particles in aqueous suspension. Each solid line indicates the best possible linear regression.

*F*_0_ and *F* are the fluorescence emission intensities in the absence and presence of quencher. *K*_sv_ denotes the dynamic quenching constant, and *V* denotes the static quenching constant. [*Q*] is the concentration of quencher. Equation (1) represents a linear function between dynamic quenching and quencher concentration, where quencher collision with the excited fluorophore (E2*) returns it to the ground state without fluorescence emission [23].  Figure 7 shows the linear S-V plots for AuNPs, which were best analyzed using (1). The *K*_sv_ for E2 is 2.6 (±0.1) × 10^4^ M^−1^ (*R* ^2^ = 0.9478), the *K*_sv_ for E2-MIP particles is 2.9 (±0.1) × 10^4^ M^−1^ (*R* ^2^ = 0.9566), and the *K*_sv_ for E2-bound NIP particles is 3.3 (±0.1) × 10^4^ M^−1^ (*R* ^2^ = 0.9678). Due to their large size, AuNPs could hardly penetrate the porous structures of E2-MIP and E2-NIP particles. Consequently, their *K*_sv_ values were in the same order of magnitude as that obtained for E2 in aqueous solution.

**Figure 8:**
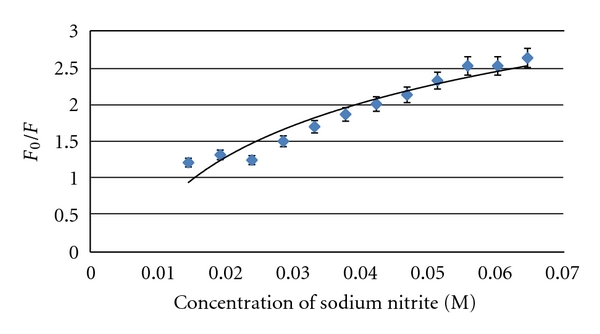
Stern-Volmer plots of *F*_0_/*F* versus concentration of sodium nitrite. Sodium nitrite was titrated stepwise into 2.5 mg/mL E2-MIPs in aqueous suspension containing 5.88 × 10^−4^ M AuNPs. Each solid line indicates the best possible regression.

The S-V plots for sodium nitrite was found to be nonlinear (with an upward-curving trend), as shown in Figure 8. Nonlinear S-V plots had been discussed by Zhao and Swager [22] as a combined result from dynamic and static quenchings. In contrast to dynamic quenching, the mechanism of static quenching involves interaction between the quencher and fluorophore to form a nonfluorescent complex [23]. Analysis using (2) obtained *K*_sv_ = 2.1 × 10^2^ M^−1^ and static quenching constant (*V*) below 1.0 M^−1^ (*R* ^2^ = 0.9220) for 2.5 mg/mL E2-MIPs in aqueous suspension containing 5.88 × 10^−4^ M AuNPs. Since MIP cavities did not facilitate complex formation between E2 molecules and nitrite anions due to space constraints, the *V* value turned out to be very small. A higher *V* value of 4.7 M^−1^ was obtained for 4.5 ppm E2 in aqueous solution, which signifies the complexation of E2 molecules with nitrite anions in the absence of steric hindrance. 

### 3.4. Determination of E2 in Water

Various concentrations (from 0.1 ppb up to 3.5 ppm) of E2 in aqueous solution were used to validate MIP particles for rapid E2 determination by fluorescence quenching. MIP particles were added in these E2 solutions to form 0.5–3.5 mg/mL suspensions. Two incubation times (5 min and 35 min) were tested to investigate binding equilibrium between E2 molecules and MIP particles. The fluorescence emission intensity from E2 specifically bound with MIP particles was determined by measuring the fluorescence emission intensities after two quenching steps, as summarized by

(3) $$\begin{array}{cc} I_{\text{E}2\;\text{inside}\;\text{MIP}\;\text{cavities}} & =I_{\text{After}\;\text{quenching}\;\text{with}\;\text{AuNps}\;}\\ & \;-\;I_{\text{After}\;\text{quenching}\;\text{with}\;\text{sodium}\;\text{nitrite}}. \end{array}$$

**Figure 9:**
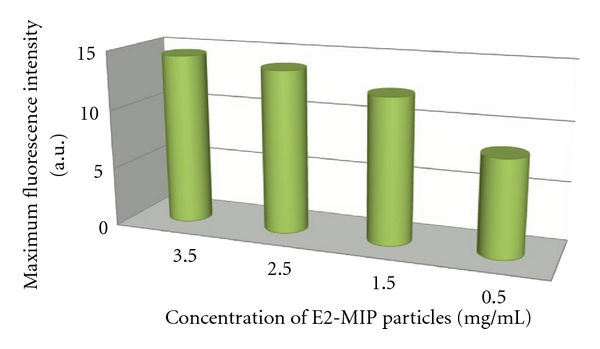
*I*_max_ for 3.5 mg/mL, 2.5 mg/mL, 1.5 mg/mL, and 0.5 mg/mL E2-MIP particles in aqueous suspension.

Essentially, the first quenching with AuNPs would eliminate the fluorescence emission from E2 molecules on nonspecific binding sites throughout the porous MIP particles. Then, sodium nitrite would quench the fluorescence emission from E2 molecules inside the specific imprinted cavities. The normalized % binding of E2 with imprinted cavities was determined as

(4) $$\text{Normalized}\;\text{\%}\;\text{E}2\;\text{binding}=\;\frac{I}{I_{\max\;}},$$

where *I* is the fluorescence intensity from E2 bound specifically inside imprinted cavities for an E2 standard solution, and *I*_max_ is the maximum fluorescence intensity from E2 bound specifically inside all imprinted cavities. Both *I* and *I*_max_ were calculated according to (3), in parallel measurements. To determine *I*_max_, E2-MIP particles were prepared by washing only with DDW to remove all nonspecifically bound E2 molecules. Normalization was deemed necessary because *I*_max_ is not linearly dependent on the concentration of E2-MIP particles in aqueous suspension, as shown in Figure 9, due to inner filter effects.

**Figure 10:**
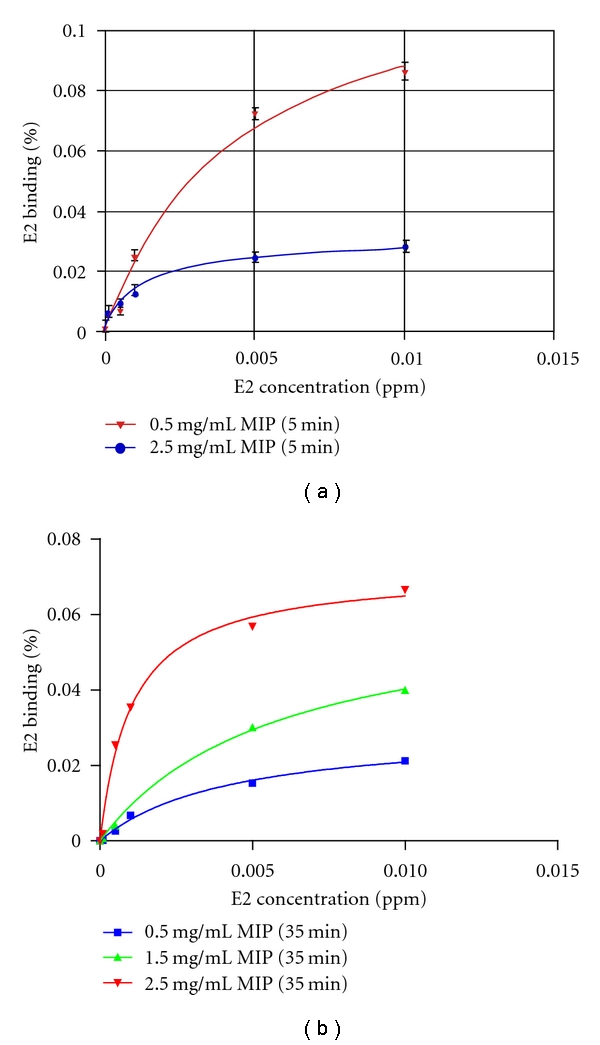
% E2 binding (with imprinted cavities) as a function of E2 concentration after incubation for (a) 5 min, and (b) 35 min (standard error bars, approximately three- to five-times the size of each data point symbol, are omitted here for the sake of clarity).

Figure 10 shows % E2 binding (with imprinted cavities) as a function of E2 concentration after incubation for (a) 5 min and (b) 35 min. A comparison of (a) with (b) indicates that % E2 binding exhibited no significant difference. This suggests that binding equilibrium was reached in 5 min or less, in agreement with the ~2 min previously reported [24]. As 0.5 mg/mL E2-MIP particles had the smallest number of imprinted cavities and hence the lowest *I*_max_ value, it produced the highest % E2 binding among the three concentrations studied. As the method involved binding of the analyte with MIP particles for the best possible selectivity (only second to natural antibodies), Scatchard plots were constructed in accordance with the following equation [25]:

(5) $$\text{\%}\;\text{E}\;\text{binding}=\;\frac{\left[\text{E}2\right]}{\left(K_{d}+\left[\text{E}2\right]\right)},$$

where *K*_*d*_ is the equilibrium binding constant. The best calibration curves, fitted using Graphpad Prism, are shown as solid lines in Figure 10. The best correlation coefficients were obtained for 2.5 mg/mL MIP particles, being *R* ^2^ = 0.9716 for 5 min of incubation and 0.9937 for 35 min of incubation.  Table 1 shows that the equilibrium binding constant (*K*_*d*_) for 2.5 mg/mL of MIP particles, using 5 min of incubation, was the highest among the three MIP concentrations studied as expected. 

## 4. Conclusion 

AuNPs (43 ± 5 nm) were efficient in quenching the fluorescence emission from E2 molecules in aqueous solution, or nonspecifically bound with porous particles. For E2 molecules specifically bound inside imprinted cavities, fluorescence quenching by sodium nitrite was successful. A rapid method (under 10 min) has been developed for E2 determination by measuring the change in fluorescence emission intensities between these two fluorescence quenching steps, using first AuNPs and then sodium nitrite. One major advantage of this method is the high selectivity of MIP particles for E2, as previously demonstrated using molecules with similar structures (estrone, ethynylestradiol) [21] and dissimilar structures (bisphenol A) [26]. Other fluorescent molecules would not interfere with the E2 determination because they can only bind nonspecifically to be readily quenched by AuNPs. Highly correlated Scatchard plots (*R* ^2^ > 0.97) serve well as a standard calibration curve. The detection limit for E2 is low, at the ultratrace level of 0.1 ppb. The method is also promising for use on a portable spectrofluorometer in field studies. Further work is underway to verify that no potential interference by common organics and anions (CO_3_^2−^, NO_3_^−^, PtCl_4_^2−^, SCN^−^, N_3_^−^ present in environmental waters) exists after an extra centrifugation step is added (to precipitate the MIP particles out for transfer into a cuvette of deionized water) before the two fluorescence quenching measurements. Optimization of the method will also be completed with testing of real sample matrices from environmental waters. 

## Figures and Tables

**Table 1:** Equilibrium binding constant (*K*_*d*_) values determined for three concentrations of MIP particles in aqueous suspension, after 5 min of incubation.

Incubation time (min)	Concentration of MIP particles (mg/mL)	*K*_*d*_ (ppm)
5 min	0.5	0.7
	1.5	1.4
	2.5	2.9
